# Gastroenteritis aggressive versus slow treatment for rehydration (GASTRO): a phase II rehydration trial for severe dehydration: WHO plan C versus slow rehydration

**DOI:** 10.1186/s12916-019-1356-z

**Published:** 2019-07-01

**Authors:** Kirsty A. Houston, Jack Gibb, Peter Olupot-Olupot, Nchafatso Obonyo, Ayub Mpoya, Margaret Nakuya, Rita Muhindo, Sophie Uyoga, Jennifer A. Evans, Roisin Connon, Diana M. Gibb, Elizabeth C. George, Kathryn Maitland

**Affiliations:** 1 Department of Paediatrics, Faculty of Medicine, St Mary’s Campus, Norfolk Place, Imperial College, London, W2 1PG UK; 2 KEMRI-Wellcome Trust Research Programme, PO Box 230, Kilifi, Kenya; 3 Mbale Clinical Research Institute, Pallisa Road, PO Box 291, Mbale, Uganda; 4 Mbale Regional Referral Hospital, Pallisa Road, PO Box 291, Mbale, Uganda; 5 Soroti Regional Referral Hospital, PO Box 289, Soroti, Uganda; 6 Department of Paediatrics, University Hospital of Wales, Heath Park, Cardiff, CF14 4XW Wales UK; 7 MRC Clinical Trials Unit at UCL 90 High Holborn, 2nd Floor, London, WC1V 6LJ UK

**Keywords:** Rehydration, Severe dehydration, Intravenous fluids, African children, Clinical trial

## Abstract

**Background:**

World Health Organization rehydration management guidelines (plan C) for severe dehydration are widely practiced in resource-poor settings, but never formally evaluated in a trial. The Fluid Expansion as a Supportive Therapy trial raised concerns regarding the safety of bolus therapy for septic shock, warranting a formal evaluation of rehydration therapy for gastroenteritis.

**Methods:**

A multi-centre open-label phase II randomised controlled trial evaluated two rehydration strategies in 122 Ugandan/Kenyan children aged 60 days to 12 years with severe dehydration secondary to gastroenteritis. We compared the safety and efficacy of standard rapid rehydration using Ringer’s lactate (100 ml/kg over 3 h (6 h if < 1 year), incorporating 0.9% saline boluses for children with shock (plan C) versus slower rehydration: 100 ml/kg Ringer’s lactate over 8 h (all ages) without boluses (slow: experimental). The primary outcome was the frequency of serious adverse events (SAE) within 48 h including cardiovascular, respiratory and neurological complications. Secondary outcomes included clinical, biochemical and physiological measures of response to treatment by intravenous rehydration.

**Results:**

One hundred twenty-two eligible children (median (IQR) age 8 (6–12) months) were randomised to plan C (*n* = 61) or slow (*n* = 61), with two (2%) lost to follow-up at day 7). Following randomisation mean (SD) time to start intravenous rehydration started was 15 min (18) in both arms. Mean (SD) fluid received by 1 hour was greater in plan C (mean 20.2 ml/kg (12.2) and 33.1 ml/kg (17) for children < 1 year and >− 1 year respectively) versus 10.4 ml/kg (6.6) in slow arm. By 8 hours volume received were similar mean (SD) plan C: 96.3 ml/kg (15.6) and 97.8 ml/kg (10.0) for children < 1 and ≥ 1 year respectively vs 93.2 ml/kg (12.2) in slow arm. By 48-h, three (5%) plan C vs two (3%) slow had an SAE (risk ratio 0.67, 95% CI 0.12–3.85, *p* = 0.65). There was no difference in time to the correction of dehydration (*p* = 0.9) or time to discharge (*p* = 0.8) between groups. Atrial natriuretic peptide levels rose substantially by 8 hours in both arms, which persisted to day 7. Day 7 weights suggested only 33 (29%) could be retrospectively classified as severely dehydration (≥ 10% weight loss).

**Conclusion:**

Slower rehydration over 8 hours appears to be safe, easier to implement than plan C. Future large trials with mortality as the primary endpoint are warranted.

**Trial registration:**

ISRCTN67518332. Date applied 31 August 2016.

## Background

Worldwide, an estimated 2.5 billion cases of acute gastroenteritis occur annually in children under 5 years. In this age group, gastroenteritis is the second biggest cause of mortality (after acute respiratory illnesses) [1, 2]. The vast majority of deaths occur in low resource settings in South Asia and sub-Saharan Africa. A large case-control study, Global Enteric Multicentre study of gastroenteritis (GEMS), conducted in Africa and Asia showed that children with moderate/severe gastroenteritis seeking care at health centres are 8.5 times more likely to die than non-gastroenteritis controls [3, 4]. A third of the fatalities occurred < 7 days following enrolment—indicating that current treatment strategies may not be working in practice and/or are poorly implemented [5].

The World Health Organization (WHO) has produced guidance on the management of dehydration in children with diarrhoea, largely based on expert opinion [6, 5]. In children (without severe malnutrition), oral rehydration solution (ORS) is recommended for children with diarrhoea with some dehydration, defined as two or more of the following: restlessness/irritability, sunken eyes, drinks eagerly/thirsty and/ or skin pinch goes back slowly (plan B). Intravenous fluids are recommended for resuscitation of children with severe dehydration, defined as the presence of two or more of the following clinical features: sunken eyes, skin pinch goes back very slowly ≥ 2 seconds (s), lethargy and/or inability to drink (under the protocol called ‘plan C’), using 100 ml/kg of Ringer’s lactate or 0.9% saline [6]. This is the approximate volume estimated to have been lost in children with 10% dehydration and is recommended to be given over 3 h (or 6 h in children < 1 year). Plan C consists of two steps in which the volume and rate of infusion are different and also differ by age group (< 1 year and ≥ 1 year). In Step 1 30 ml/kg over 30 min (or 1 h if < 1 year) and then in Step 2 70 ml/kg over 2.5 h (or 5 h if < 1 year). For children presenting with shock (defined as the presence of all three of weak and fast pulse, temperature gradient and capillary refilling time > 3 s), WHO also recommends initial fluid boluses given for shock (i.e. up to 3 boluses of 20 ml/kg of normal (0.9%) saline given as rapidly as possible) followed directly by Step 2, i.e. 90–130 ml/kg. These management guidelines are recommended in resource-poor settings, despite no formal testing in a clinical trial [7]. A review of the evidence underpinning WHO management guidelines in 2012, with regard to shock and rehydration management, focused principally on the type of fluid for resuscitation but did not consider the rate or volume [5].

### Evidence from trials, reviews and audits

In the phase III FEAST randomised controlled trial, fluid bolus therapy was compared with maintenance fluids in children with signs of shock and febrile illness, which showed 3.3% higher absolute mortality in children randomised to fluid bolus therapy compared with maintenance only (control). A subsequent analysis of the terminal clinical event revealed that the excess mortality in children enrolled in the fluid bolus arms was attributable to cardiovascular collapse rather than neurologic or respiratory events [8]. Children with acute gastroenteritis, severe malnutrition and fluid loss due to other causes were excluded from the trial [9]. The FEAST trial also raised concerns regarding the safety of rapid intravenous rehydration therapy in other settings and other common illnesses requiring aggressive fluid such as acute gastroenteritis.

A systematic review of intravenous rehydration worldwide found only 3 trials (*n* = 464) comparing rates of rehydration; none were conducted in resource-poor settings [10]. There were no deaths in any of the reported trials but a pooled analysis suggested longer time-to-discharge and higher readmission rates in the rapid rehydration arms [10, 7]. Emerging data on outcomes from acute gastroenteritis across a network of Kenya hospitals involving 1211 children with severe dehydration who were managed with the current WHO plan C found that in-hospital mortality was 8% in those with severe dehydration and increased to 34% if this was complicated by shock [11]. In summary, the current evidence base for plan C has been judged to be of very low quality as it relies on expert option.

Given the information provided by the FEAST trial and the high early mortality for acute gastroenteritis with severe dehydration, we conducted a phase II trial investigating the impact of different rates of rehydration on clinical and physiological outcomes.

## Methods

### Study design and treatment protocol

We conducted a multi-centre, open-label phase II randomised controlled trial comparing WHO standard (fast or aggressive) versus slow (experimental) intravenous rehydration of children admitted to hospital with gastroenteritis and severe dehydration. Eligible children from three clinical centres in Eastern Uganda (Mbale and Soroti Regional Referral Hospitals) and Kenya (Kilifi County Hospital) were randomly assigned on admission to hospital (ratio 1:1) to receive either (1) WHO plan C, 100 ml/kg over 3 (≥ 1 year) or 6 h (< 1 year), plus additional boluses for treatment of shock, or (2) slower rehydration, 100 ml/kg over 8 h regardless of age, without fluid boluses. To assist with the accurate administration of intravenous fluid volumes and rates, the study used gauged burettes and infusion sets to ensure adherence to the protocol. At the time of the trial, Kenya had introduced rotavirus vaccination for young infants but Uganda had not. During the period of study, there were no reported epidemics of cholera in either location.

### Study population

#### Screening procedures

We aimed to enrol 120 children, aged 60 days to 12 years, with severe dehydration secondary to gastroenteritis at admission to the paediatric ward. Dedicated trial clinicians and nurses screened potentially eligible children. Children were eligible if they had a history of gastroenteritis (> 3 loose stools/day) and signs of severe dehydration (as per WHO definition, two or more of the following: unable to drink or AVPU <A, with sunken eyes and reduced skin pinch (> 2 s) and/or an inability to take or retain oral fluids), with or without shock. Hypovolaemic shock was defined by the recent 2016 WHO ETAT criteria, a patient with all of the following: cold peripheries with a weak and fast pulse (rate not specified) and a capillary refill time more than 3 s. We excluded children with severe malnutrition (kwashiorkor or MUAC < 11.5 cm), diarrhoea lasting more than 14 days, known congenital or rheumatic heart disease and absence of a parent or guardian willing/able to give consent.

### Outcome measures

The primary outcome was the frequency of fluid-related serious adverse events (including mortality, cardiovascular collapse, suspected raised intracranial pressure, pulmonary oedema) within 48 h. Secondary outcomes included time to correct signs of severe dehydration, time to tolerate oral fluids/feeds and time to discharge, dysnatraemia (defined as < 135 mmol or > 145 mmol/L) [7] at 8 h and readmission to hospital (within 7 days post-discharge).

### Study procedures

#### Screening and randomisation

Children with suspected severe dehydration based on presenting symptoms were clinically assessed for severity and other exclusion criteria. Once eligibility was confirmed, authorised trial staff approached parents/guardians to invite their child to take part in the trial. Where prior written consent from parents/legal guardians could not be obtained, ethics committees approved parental verbal assent and deferred written informed consent as soon as practicable [12]. Otherwise, informed written consent was obtained from parents or guardians before randomisation. The randomisation list and envelopes were prepared by an independent data manager in KWTP Kilifi. The lists were not available to investigators and were stratified by clinical centre. The schedule for each centre contained a list of trial numbers and the randomly allocated intervention. The treatment allocation (plan C versus slow intravenous rehydration) was kept in numbered, sealed opaque envelopes. The cards were numbered consecutively and opened in numerical order.

### Sample size

A formal sample size was not calculated. We aimed to study 120 children (60 in each arm) to provide sufficient pilot data (clinical and physiological) on the main outcomes given the timeframe of the study whilst balancing against exposing children to a therapeutic intervention (rate) for which there are limited data. We have used similar sample sizes in the past in fluid resuscitation studies (comparing rates and different types of fluids that informed the design of FEAST) [13, 14].

### Clinical monitoring and study assessments

Following consent and randomisation, the patients were commenced on the intravenous rehydration protocol as detailed in Table 1. Clinical and haemodynamic responses were monitored at 1, 2, 4, 8, 12 h and then daily until discharge. At each clinical assessment, children were assessed for pre-specified serious adverse events of interest (new-onset seizures or worsening conscious level (neurological), signs of pulmonary oedema, cardiac failure or cardiovascular collapse post-randomisation). Blood samples were routinely collected at admission, 8 h and 24 h and stored for future batch processing. This included standard biochemistry, cardiac biomarkers (cardiac troponin I (cTnI); A type and B type natriuretic peptides (ANP and BNP respectively)) at admission, 8 h and 24 h. Stool samples were not collected so no data are available on the pathogenic aetiology of gastroenteritis in the trial population**.**

**Table 1:** Trial treatment arms

Age	WHO Plan C		Slow arm	
	No shock	With WHO shock*	No shock	With shock
< 12 months	Step 1 30 ml/kg over 1 h Step 2 70 ml/kg over 5 h	Resuscitate: Up to 3 × 20 ml/kg bolus then to step 2 70 ml/kg over 5 h	100 ml/kg over 8 h	100 ml/kg over 8 h with No additional boluses
> 12 months	Step 1 30 ml/kg over 30 min Step 2 70 ml/kg over 2.5 h	Resuscitate: Up to 3 × 20 ml/kg bolus then to step 2 70 ml/kg over 2.5 h	100 ml/kg over 8 h	100 ml/kg over 8 h No additional boluses

*Shock defined as presence of all three of the following: Weak and fast pulse, temperature gradient and prolonged capillary refill time > 3 s (10)

### Cardiac biomarker methodology

Blood was collected in tubes with ethylene diamine tetraacetic acid (EDTA) anticoagulant, centrifuged at 4 °C and a volume of 500 μL aliquoted and frozen at − 80^0^. Plasma samples were batch-processed and cardiac biomarkers quantified using standard human enzyme linked immunosorbent assays (ELISAs) (ANP and BNP, Thermofisher Scientific and cTnI, Abcam®).

### Serious adverse events

Serious adverse events (SAE) were reported on a standardised SAE form and sent to the Clinical Trials Facility, Kilifi, Kenya, and to local ethics board and were monitored against source documents by independent monitors. The SAE forms the local clinician SAE monitor blinded all SAES to intervention strategy before sending these to the Endpoint Review Committee (ERC) who reviewed all deaths, neurologic events and other SAEs blinded to treatment arm. The classification of suspected pulmonary oedema and cardiovascular collapse used the same definitions as reported in FEAST paper reporting modes of death [8].

### Further management

Following correction of dehydration (based on a review of WHO clinical signs and observations), children were assessed for their ability to take oral rehydration or feeds. Children who were able to take and retain oral fluids/feeds and who were in neutral or marginally positive fluid balance (both input and output were measured) were considered as satisfactorily rehydrated. For the purposes of protocol adherence, each child completed their allocated intravenous fluid hydration regimen regardless of when they were satisfactorily rehydrated, as WHO recommends to complete rehydration first before re-evaluating [4]. Once able to tolerate oral fluids, children were offered oral rehydration fluids alongside their intravenous rehydration regimen. Each child had an input-output fluid monitoring chart, which included urinary catheter volumes (where parents consented) and diaper weights, designed to document the volumes that children in both arms were drinking and retaining as well as defining clinical end-points. If a child developed suspected signs of fluid overload, intravenous fluids were stopped and if there were signs of pulmonary oedema, clinicians were permitted to administer intravenous furosemide (1–2 mg/kg) and supplemental oxygen (if saturations < 90% by pulse oximetry). When stable, further fluid management was administered orally (or via nasogastric tube if the child was unable to take fluids orally). If there were ongoing significant gastrointestinal fluid losses with persisting dehydration, the protocol permitted one repeat of rehydration regime as per their randomised arm. Only after this were clinicians free to personalise additional fluid management to take account of input/output.

Children returned for follow-up on day 7 for a clinical assessment, weight and vital sign observations, a further blood sample (for biochemistry and cardiac biomarkers) and urine sample for urinary electrolytes. These assessments of hydration (in children without any on-going losses or intercurrent illness) served to validate inpatient assessments.

### Data management and statistical analysis

All clinical and laboratory data was recorded in case report forms (CRFs) with a unique identifier. Data was entered onto Open Clinica. All statistical analyses were documented a priori in the statistical analysis plan and were undertaken using intention-to-treat. The risk ratio and risk difference were calculated to compare proportions reaching the primary outcome between arms. Time-to-event secondary outcomes were analysed using Kaplan Meier plots and log rank tests. Proportions of children with dynstraemia at 8 h were compared with a *χ*^2^ test.

## Results

### Study patients

**Fig. 1:**
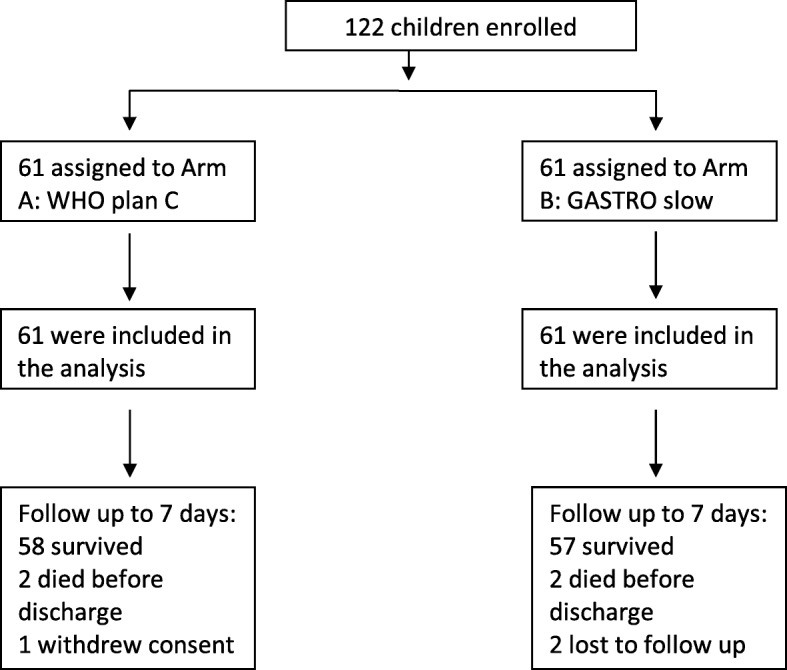
Trial flow

One hundred twenty-two children were randomised from 9 January 2017 to 15 February 2018 across the three sites (69 at Mbale, 45 at Soroti and 8 at Kilifi), with 61 allocated to each arm. All children included in the trial met the eligibility criteria (trial flow: Fig. 1). Baseline characteristics were broadly balanced between arms, although some differences were present as expected given small sample size. The median age was 8 months (IQR 6–12); 70% were under 12 months of age. On admission, 69 children (57%) were febrile (axillary temperature > 37.5C), 86 (70%) had very sunken eyes and 66 (54%) had severely decreased skin turgor. Most had altered conscious level with only 23% of patients reported as alert, 26 (21%) were prostrated and 3 were comatosed and 11% (14) had signs of respiratory distress (Table 2).

**Table 2:** Baseline characteristics

	WHO plan C	GASTRO slow	Total
Female, *n* (%)	23 (38)	27 (44)	50 (41)
Age (months), median (IQR)	9 (6–12)	8 (6–12)	8 (6–12)
Weight (kg), median (IQR)	7.7 (6.8, 8.3)	7.3 (6.8,8.3)	7.5 (6.7–8.3)
Axillary temperature (°C), median (IQR)	37.6 (37.0, 38.2)	37.8 (37.2, 38.3)	37.7 (37.0, 38.2)
< 36, *n* (%)	1 (2)	1 (2)	2 (2)
> 37.5, *n* (%)	32 (52)	37 (61)	69 (57)
Sunken eyes, *n* (%)			
Slightly sunken	17 (28)	18 (30)	35 (29)
Very sunken	44 (72)	42 (69)	86 (70)
Decreased skin turgor^a^, *n* (%)	32 (52)	34 (56)	66 (54)
Heart rate, median (IQR)	145.0 (138.5, 159.0)	149.5 (140.5, 160.5)	148 (139, 160)
Bradycardia (< 80 bpm), *n* (%)	0 (0)	0 (0)	0 (0)
Severe tachycardia^b^, *n* (%)	7 (11)	11 (18)	18 (15)
Systolic blood pressure, median (IQR)	90 (85, 95)	89 (85, 96.5)	89 (85, 96)
Moderate hypotension, *n* (%)^c^	2 (3)	5 (8)	7 (6)
Severe hypotension, *n* (%)^d^	1 (2)	0 (0)	1 (1)
Capillary refill time, median (IQR)	1 (1, 1)	1 (1, 1)	1 (1, 1)
≥ 3, *n* (%)	5 (8)	3 (5)	8 (7)
No. with weak pulse, *n* (%)	6 (10)	7 (11)	13 (11)
No. with temperature gradient, *n* (%)	11 (18)	10 (16)	21 (17)
Respiratory rate, median (IQR)	40 (37, 48)	44 (40, 50)	43 (38, 49)
Fast breathing, *n* (%)	2 (3)	3 (5)	5 (4)
Respiratory distress, *n* (%)	5 (8)	9 (15)	14 (11)
Oxygen saturation, median (IQR)	98 (97, 99)	98 (97, 98)	98 (97, 99)
Conscious level, *n* (%)			
Alert	12 (20)	16 (26)	28 (23)
Lethargic	35 (57)	30 (49)	65 (53)
Prostrate	13 (21)	13 (21)	26 (21)
Coma	1 (2)	2 (3)	3 (2)
No. with fits or convulsions, *n* (%)	1 (2)	0 (0)	1 (1)

^a^Recoil from skin pinch > 2 s

^b^Defined as heart rate > 180 for age < 12 months; > 160 for age 12–60 months; > 140 for age ≥ 60 months

^c^Moderate Hypotension defined as: if < 12 months = 50-75 mmHg; 12 months to 5 yrs = 60-75 mmHg; > 5 yrs = 70-85mmHg

^d^Severe hypotension defined as: if < 12 months ≤ 50mmHg; 12 months to 5 yrs ≤ 60mmHg, > 5 yrs ≤ 70mmHg

Children at admission had evidence of markedly deranged biochemistry (Table 3). Overall, 24/103 (23%) had hyponatraemia (sodium < 135 mmol/L); 38 (37%) had hypernatremia (sodium > 145 mmol/L) of whom 27 (26%) had severe hypernatraemia (sodium > 150 mmol/L). As expected, there was a significant number of children with high urea, 52 (50%), and/or high creatinine (> 74 mmol/L). After admission, only one child (plan C) developed hypoglycaemia (glucose < 3 mmol/L) requiring correction.

**Table 3:** Admission, 8 hour and 24 hour biochemistry

	Plan C	GASTRO slow	Total
Admission laboratory			
*N*	53	50	103
Sodium, median (IQR)	139 (132, 149)	143 (137, 156)	142 (136, 154)
Hyponatremia (< 135 mmol/L), *n* (%)	16 (30)	8 (16)	24 (23)
Hypernatremia (> 145 mmol/L), *n* (%)	15 (28)	23 (46)	38 (37)
Severe hypernatraemia > 150 mol/L, *n* (%)	11 (21)	16 (32)	27 (26)
Hypokalaemia (< 3.5 mmol/L), *n* (%)	11 (21)	8 (16)	19 (18)
High creatinine (> 74 mmol/L), *n* (%)	11 (21)	12 (24)	23 (23)
High urea (> 6.4 mmol/L), *n* (%)	26 (49)	26 (52)	52 (50)
Glucose, median (IQR)	5.4 (4.9, 6.3)	5.3 (4.4, 6.9)	5.4 (4.7, 6.8)
Lactate^a^, median (IQR); *N*	1.2 (1.0, 2.1); 29	1.3 (1.1, 1.7); 27	1.3 (1.0, 1.7); 56
High lactate > 3 mmol/l, *n* (%)	4 (14)	0 (0)	4 (7)
At 8 hours			
*N*	50	52	102
Sodium, median (IQR)	142 (135, 147)	142 (138, 148)	142 (136, 148)
Hyponatremia (< 135 mmol/L), *n* (%)	12 (24)	6 (12)	18 (18)
Hypernatremia (> 145 mmol/L), *n* (%)	17 (34)	19 (37)	36 (35)
Severe hypernatraemia > 150 mol/L, *n* (%)	7 (14)	10 (19)	17 (17)
At 24 hours			
*N*	41	45	86
Sodium, median (IQR)	142 (138, 154)	143 (138, 156)	143 (138, 155)
Hyponatremia (< 135 mmol/L), *n* (%)	7 (17)	5 (11)	12 (14)
Hypernatremia (> 145 mmol/L), *n* (%)	16 (39)	18 (40)	34 (40)
Severe hypernatraemia > 150 mol/L, *n* (%)	12 (29)	16 (36)	28 (33)

^a^Soroti sites were unable to do lactate tests

### Adherence to protocol

**Fig. 2:**
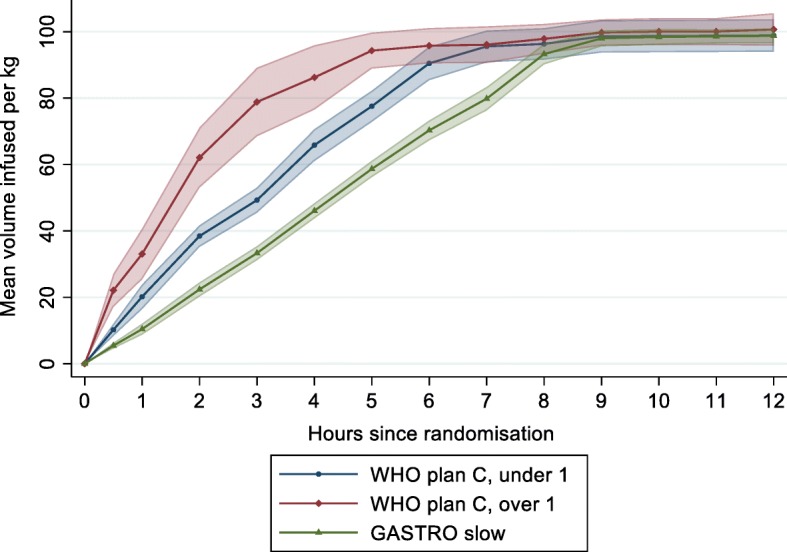
Mean volumes of fluid received during the first 12 h from randomisation. Mean volume (in millilitres per kilogramme) and 95% CI calculated at each time point (30 min, 1 h then hourly until 12 h)

In the plan C arm, 2 children met the criteria for shock on admission and received boluses (1 child had one bolus of 20 ml/kg and 1 child had 2 × 20 ml/kg boluses) and 1 child in the slow arm met the shock criteria and did not receive a bolus (Table 4). The time taken to start fluids was similar in both arms with a mean 15 min (sd 18) from randomisation. At 1 h, children < 1 year had received a mean 20.2 ml/kg (sd 12.2) and children ≥ 1 year received 33.1 ml/kg (sd 17) in plan C arm and children in the slow arm received a mean 10.4 ml/kg (sd 6.6). By 8 h, the mean volume received was very similar between arms (96.3 ml/kg (sd 15.6) children < 1 year and 97.8 ml/kg (sd 10.0) for children ≥ 1 year in plan C, and 93.2 ml/kg (sd 12.2) in slow arm) (Table 2, Fig. 2). Non-adherence to protocol occurred in one child in the plan C arm who died before receiving the total amount of fluids and 2 patients in the slow arm received their fluids over a longer period of time (24 and 28 h respectively) than described in the protocol. An additional 2 patients (one in each arm) had an incomplete recording of the fluid volume received.

**Table 4:** Volumes of fluid received during the first 12 h of admission

		WHO plan C		GASTRO slow
		Under 1 year	1 year or over	All ages
Patients, *n*		42	19	61
Number of patients in shock		1	1	1
Number of patients who received initial bolus		1	1	0
Total volume of bolus fluid received	Mean	20	40	0
Total volume of fluid received (ml/kg) up to:				
30 min	Mean (sd)	10.2 (6.1)	22.1 (11.1)	5.4 (3.5)
	Median (IQR)	14.9 (7.5, 15)	30 (15, 30)	6.2 (3.2, 6.3)
1 h	Mean (sd)	20.2 (12.2)	33.1 (17.0)	10.4 (6.6)
	Median (IQR)	30 (15, 30)	30 (30, 44)	12.5 (6.3, 12.6)
3 h	Mean (sd)	49.3 (12.5)	78.8 (23.0)	33.3 (8.4)
	Median (IQR)	44.1 (44, 58)	86.1 (58, 100)	37.4 (25.1, 37.6)
6 h	Mean (sd)	90.5 (16.7)	95.8 (11.8)	70.3 (11.9)
	Median (IQR)	100 (86, 100)	100 (100, 100)	74.8 (62.9, 75.2)
8 h	Mean (sd)	96.3 (15.6)	97.8 (10.0)	93.2 (12.2)
	Median (IQR)	100 (100, 100)	100 (100,100)	100 (87.7100)
12 h	Mean (sd)	98.9 (16.2)	100.7 (10.9)	98.7 (8.6)
	Median (IQR)	100 (100, 100)	100 (100, 100)	100 (100, 100)

### Primary outcomes

In the plan C arm, 3 patients (5%) had a serious adverse event (two cardiovascular collapse (resulting in death) and one status epilepticus (resolved) within 48 h of randomisation compared with 2 (3%) (suspected pulmonary oedema and cardiovascular collapse (both resulting in death) in the slow arm (risk ratio 0.67, (95% CI 0.12, 3.85); risk difference − 1.6%, (95% CI − 8.7%, 5.4%); *p* = 0.65). There was 1 additional SAE (convulsions) in the slow arm that occurred at 101 h from randomisation, which resolved without other complications (Table 5). Only one of the SAEs was judged to be probably related to the study fluid (in the plan C arm), with the other 5 unlikely to be related (clinical details summarised in Table 6). Overall mortality (to hospital discharge) was 4/122 (3.3%).

**Table 5:** Serious adverse events recorded during the trial

	WHO plan C *n* = 61	GASTRO slow *n* = 61
Number of SAEs	3 (4.9%)	3 (4.9%)
Outcome of SAE		
Resolved	1	1
Died	2 (3.3%)	2 (3.3%)
Relationship to study fluid		
Unlikely to be related	2	3
Probably related	1	0
Nature of event		
Pulmonary oedema^a^	0	1
Cardiovascular collapse^b^	2	1
Other:		
Status Epilepticus (child with severe malarial anaemia)	1	0
Convulsions	0	1

^a^Development of hypoxia (PaO2 < 90%) with chest signs (crepitations or indrawing)

^b^Signs of shock at the point of demise: severe tachycardia or bradycardia plus one of prolonged CRT > 2 s, cold peripheries or low systolic blood pressure

**Table 6:** Serious adverse events: clinical details

Number	Site	Arm	Event type	Outcome	Relationship*	Additional details
GAS230	Mbale	Plan C	Convulsions	Resolved	Unlikely	Lethargic at admission and suffered repeated seizures/status (controlled by IV diazepam) after admission leading to worsening conscious level. Previous history of seizures prior to admission (in this illness). Severe malaria anaemia: received 1 unit of whole blood.
GAS263	Mbale	Slow	Suspected Pulmonary oedema	Fatal	Unlikely	Severe pneumonia and gastroenteritis. Left-sided pleural effusion on x-ray. Lethargic at admission. Had worsening difficulty in breathing needing supplemental oxygen. Died about 36 h into admission.
GAS002	Kilifi	Plan C	Cardiovascular collapse/heart failure	Fatal	Probable	Comatose, hypoglycaemic and in shock at admission. Fluid boluses x 2: post-resuscitation echo showed “worsening biventricular function, tricuspid regurgitation and right atrial enlargement” treated with frusemide intravenously. Died 13 h into admission: cause of death: heart failure.
GAS003	Kilifi	Plan C	Cardiovascular collapse	Fatal	Unlikely	Severe pneumonia and gastroenteritis and had prolonged capillary refilling and a weak pulse at admission. Required supplemental oxygen at admission but did get a bolus. Died 1-h post-admission following cardiovascular collapse. Blood culture: *Haemophilus influenzae*.
GAS004	Kilifi	Slow	Convulsions	Resolved	Unlikely	Comatose and hypoglycaemic at admission. Suffered seizures on day 3 of admission. Admission history suggested seizures (in this illness) prior to admission. Seizures were controlled with I.V phenobarbitone
GAS007	Kilifi	Slow	Cardiovascular collapse	Fatal	Unlikely	Comatose at admission, very sick needing supplemental oxygen (saturations 52%) by mask and had hepatomegaly 4 cm BCM. Suffered seizures following admission which were controlled with I.V phenytoin. Died about 10 h into admission following cardiac arrest that was preceded by respiratory arrest.

*By attending clinician

### Secondary outcomes

**Fig. 3:**
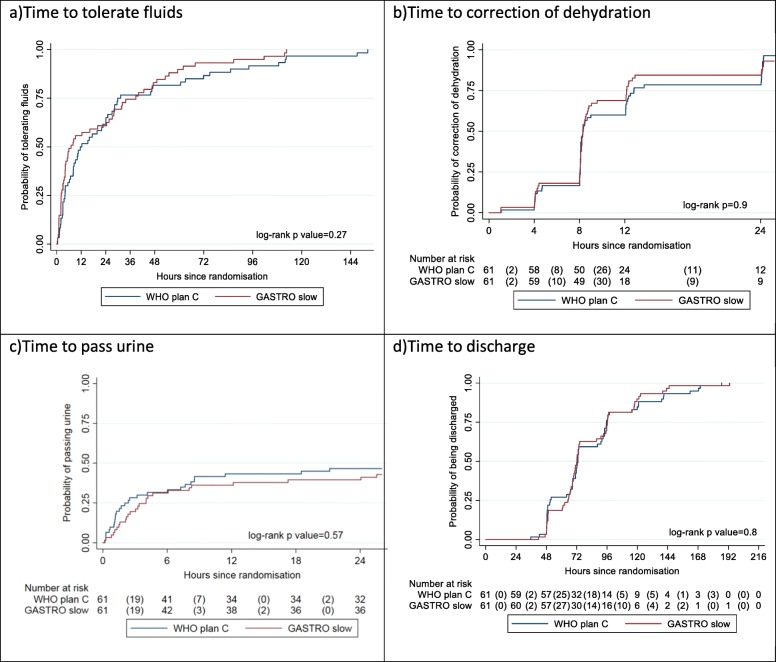
Secondary outcomes

The median time to tolerate fluids was 6.5 h (IQR 2.2–36.3) in the slow arm and 11.9 h (IQR 4.0–30.6) in plan C arm; this difference was not statistically significant (*p* = 0.27). There was no evidence of a difference in time to correction of dehydration (*p* = 0.9) nor time to discharge (*p* = 0.8) (Fig. 3). Only 54/122 patients (44%) had a record of passing urine within 24 h of starting fluids, likely due to a large number of parents not consenting for their child to be catheterised. Using the available data, there was no evidence of a difference in time to pass urine (*p* = 0.57) (Fig. 3). There were no readmissions within 7 days in either treatment arm. Vital status at 7 days was ascertained on 60 children in the plan C arm and 59 in the slow arm (Fig. 1). Dysnatraemia at 8 h was present in 29 (58%) children in the plan C arm compared to 25 (48%) children in the slow arm (*p* = 0.32) (Table 3).

### Cardiac biomarkers

**Fig. 4:**
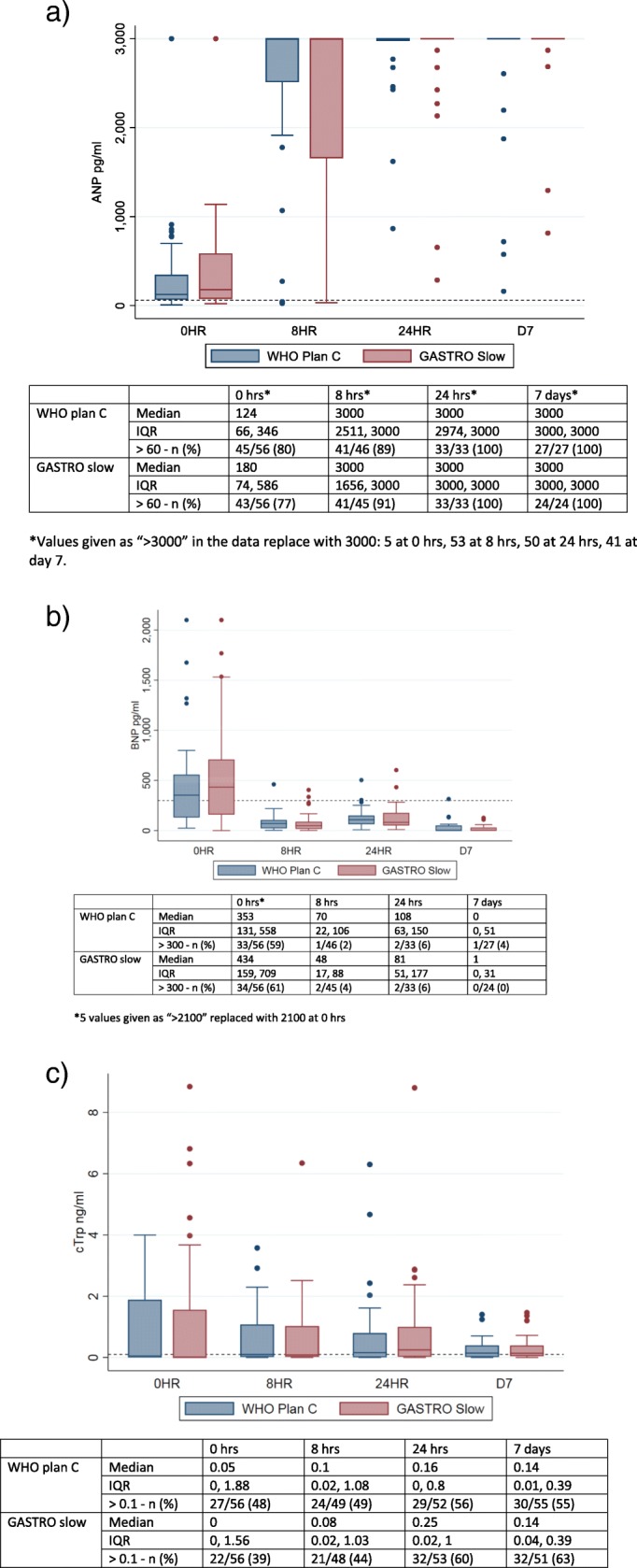
Cardiac biomarkers over time in children receiving plan C and slow rehydration. **a** Atrial natriuretic peptide (ANP). **b** Brain natriuretic peptide (BNP). **c** Cardiac troponin (cTrp)

We also aimed to evaluate the effects of volume loading (stretch) and myocardial injury by measuring cardiac troponin [15] and natriuretic peptides [16, 17]. Figure 4 summarises the median and interquartile ranges of each of the three cardiac biomarkers by randomisation arm and over time (0, 8, 24 h and 7 days). BNP levels were slightly elevated in more than half of the children in both groups at admission, returning to normal levels by 8 h. Cardiac troponin levels increased from admission and remained raised at 8 h, 24 h and day 7, but were still mainly within physiological limits. From relatively normal range at admission, there were substantial ANP levels rises at 8 h in both arms, which persisted to day 7. Overall, there were no significant differences in the ANP distribution at 8 h (Kolmogorov-Smirnov tests *p* = 0.6). We were not able to perform non-invasive monitoring of myocardial function.

### Assessment of degree of dehydration

At day 7, we reviewed the weight of 115 children and compared this to weight (using the same Salter weighing scales on both occasions) at admission (pre-rehydration) to retrospectively assess the degree of dehydration at presentation **(**Tables 7 and 8). Day 7 weight was chosen owing the previous problems with poor reliability of an in-patient assessment of weight (see the ‘Discussion’ section). Overall, day 7 weights suggested that 52 (45%) had between 0 and 5% dehydration at admission and only 33 (29%) could be retrospectively classified as severely dehydration (≥ 10%). There were few differences in the number of features of dehydration in the 0–5% and 5–10% categories and only marginally more signs of dehydration in those with severe dehydration (as indicated by ≥ 10% percentage change in weight at day 7).

**Table 7:** Percentage of weight loss at admission (compared to day 7 weight)

	WHO plan C	GASTRO slow	Total
N	58	57	115
Mean (sd)	6 (6)	7 (6)	7 (6)
Median (IQR)	6 (2, 11)	5 (3, 10)	6 (2, 11)
0–5%, *n* (%)	24 (41)	28 (42)	52 (45)
5–10%, *n* (%)	17 (29)	13 (23)	30 (26)
10%, *n* (%)	17 (29)	16 (28)	33 (29)

**Table 8:** Signs of dehydration and change in weight

	0–5%	5–10%	≥ 10%	Total
*N*	52	30	33	115
Lethargy or unconsciousness, *n* (%)	42 (80)	24 (80)	29 (88)	95 (83)
Unable to drink, *n* (%)	38 (73)	24 (80)	25 (76)	87 (76)
Sunken eyes, *n* (%)	52 (100)	29 (97)	32 (97)	113 (98)
Slow skin pinch, *n* (%)	32 (62)	20 (67)	27 (82)	79 (69)
Number of symptoms, *n* (%)				
2	11 (21)	6 (20)	3 (9)	20 (17)
3	22 (42)	11 (37)	13 (39)	46 (40)
4	19 (37)	13 (43)	17 (52)	49 (43)

## Discussion

In this clinical trial involving 122 children with severe dehydration due to gastroenteritis, we have shown that a simpler protocol involving slower rehydration with 100 ml/kg over 8-h irrespective of age was as safe as the 2-stage age-specific WHO plan C protocol. Correction of dehydration and time to tolerate oral fluids and time to discharge was similar in both arms. Overall, mortality in this phase II trial was substantially lower (3.3%) than previous reports [4, 11] and few other adverse events were recorded.

One of the additional objectives of the trial was to assess adherence to the rehydration protocol and other challenges for implementation rehydration. Whilst current management guidelines (WHO plan C) are thought to be widely practiced in African hospitals, it became apparent when conducting the training for the clinical trial that clinicians prior to the trial were not routinely able to adhere to plan C for a number of reasons. First, the administration of accurate volume and rates of infusion were found to be challenging to hospitals where gauged burettes or infusion sets are routinely unavailable. Second, the two-step plan C involving a fast infusion followed by slower intravenous replacement at two different rates and volumes for two age groups were confusing and thus difficult to recall and implement accurately in practice [18]. Finally, the WHO treatment guideline for the child with severe dehydration presenting with shock [6] is rather opaque on whether fluid boluses (20 ml/kg of normal saline given as fast as possible and repeated up to two times if shock persists) are given in addition to rehydration (thus, the maximum volume a child could receive would be 160 ml/kg) in 4–6 h. We found initially navigating and executing the plan C guideline a challenge to teach and implement, yet the trial teams, as a result of recurrent training throughout the trial, were able to demonstrate adherence to the guideline in the study arm.

**Fig. 5:**
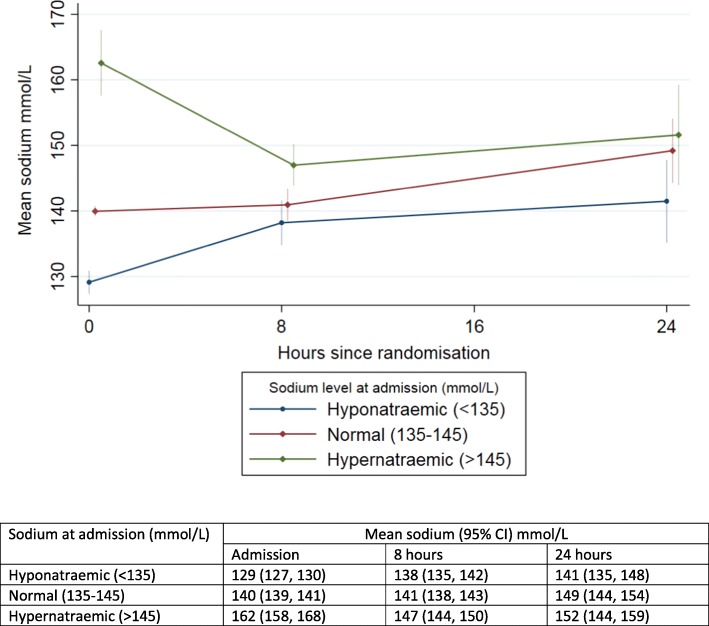
Mean (95% CI) of sodium levels over 24 h by study arm in children categorised as hyponatraemic, normomatraemic and hypernatraemic at admission

In the design of the trial, when considering secondary outcomes, we aimed to use guideline recommendations for assessing the reversal of dehydration. First, we aimed to check on whether the clinical signs for severe dehydration accurately predict the degree of dehydration. We based this on the gold standard looking at the percentage change in weight from admission to day 7, rather than weight at discharge since this time point has not been reliable previously [19, 20]. We found that although the trial was targeting children that were assumed to have 10% or more dehydration, our data suggests this was present in only 33 (29%), with 30 (26%) having a deficit of > 5- < 10% and nearly half with only modest deficit (1–5%). There have been several systematic reviews covering the issue of the specificity of all the available dehydration scores; nearly all perform poorly in practice [21] including the WHO scoring. Of note during initial rehydration, at 8-h most children remained clinically dehydrated, based on the same signs, but were not re-prescribed intravenous rehydration (even though it is recommended to start plan C again). In order to address the lack of specificity of these signs we added two additional parameters that are also considered as rehydration goals and/or adverse consequences. We selected time to pass urine as a more reliable marker of perfusion (as it is specified or required as an endpoint for management of shock or hydration status). We also included dysnatraemia, as this has been used in a number of other clinical trials as a safety endpoint (defined as a sodium level outside the normal range (135-145 mmol/L) [7]. However, we found, on stored samples, batch-analysed at the end of the trial, that quite a number had haemolysis (noted at the time of storage in – 80 °C freezers) and many had extreme sodium and chloride values at admission and 8 h which could not be rechecked for verification. We thus excluded samples with visual evidence of haemolysis (that also had extreme potassium values) and those with extreme values (sodium > 240 mmol/L and creatinine (< 20 mmol/L). However, we do note that severe hypernatraemia can also cause rhabdomyolysis, which would also result in similar findings as haemolysis (as well as dark urine) [22]. Discussions with local trial teams indicated that intravenous access for cannulation and blood draws were exceptionally difficult owing to the extreme volume depletion in many children. Moreover, our finding of exceptionally high sodium level (and chlorides) in some children is consistent with hypernatraemic dehydration, and with a history of parents rehydrating children with high-sugar energy fizzy drinks (containing 54–62 g glucose/500 ml). This practice has been reported on by previously as being a major risk factor for hypernatraemic dehydration, as well as young infants receiving only breast milk [5]. Hypertonic or hypernatraemic dehydration (defined as a sodium level > 150 mmol/L) occurs when water loss is much greater than sodium loss and usually often presents with different clinical signs (‘doughy’ skin in an irritable child and full fontanelle in an infant) [23] and is generally recommended to be managed using a separate slow rehydration protocol [24]. The current WHO rehydration Plan C protocol recommends that children with severe isotonic or hypotonic dehydration receive fast rehydration to restore intracellular sodium and water losses but makes no recommendations for rehydration of children with hypernatraemic dehydation. The most conservative estimate, when most haemolysed and extreme values were excluded, suggests that 26% of children with gastroenteritis in Eastern Uganda present with hypernatraemic dehydration (sodium > 150 mmol/L). Nevertheless, outcomes for both arms were similar with few children developing neurological complications (one of the risks of hypertonic dehydration) [23, 25] and mortality was much lower that pre-trial estimates [11]. On the whole sodium levels tended to normalise by 8- and 24-h in those presenting with hyponatraemia, normonatraemia and hypernatraemia, with little difference between the study arms (Fig. 5).

One other challenge we met was incomplete data on time to pass urine. During the consent process, we asked parents whether we were able to catheterize children; however, by 24 h, we only had data on 54/122 patients (44%) that had passed urine despite most having resolved features of dehydration and tolerating oral fluids and feeds (Fig. 3). We suggest that this was due to the fact that parents did not accept urinary catheterization (not specifically recorded in the case report form) but consented to enrolment into the trial. Alternative approaches to an 8-hourly assessment of urine output included estimating urine output from weighing nappies (diapers) or the use of urine collections bags (specifically in females) were not viable options in children with on-going diarrhoeal losses.

One hypothesis, to explain the excess mortality and mode of death observed in the FEAST trial is that cardiovascular collapse may occur as a result of right atrial stretch following aggressive fluid loading, and could be predicted by myocardial performance indices, raised ANP (atrial natriuretic peptide) [26] and subsequent urinary sodium excretion. ANP is a cardiac hormone that is released in response to atrial stretch [27]. In an ovine model of sepsis, a 14-fold rise in ANP levels was seen in experimental endotoxaemia and further rise with fluid resuscitation [28] but not in the control (no bolus arm). However, in that study, the rise was transient and peaked just after volume loading with a fluid bolus before declining. In contrast, the ANP profile in this cohort of dehydrated children rose from admission to 8 h and remained persistently high at day 7.

BNP is released in response to ventricular stretch and has been shown to have diuretic, natriuretic and vasodilator properties in published literature [16] . A study by Westerlind et al. (2008), reported the highest ANP and BNP values in children with systolic dysfunction and volume overload [29]. It has also been reported to be high in conditions such as sepsis [30, 31]. Price et al. (2006) reported a higher risk of hospitalisation and mortality in children with systolic dysfunction and elevated BNP levels [32]. In this cohort, BNP levels were highest at admission in both groups with severe dehydration, which was not accounted for by high creatinine level in this with high BNP, then decreased over the first 8-h despite fluid administration, but later increased at 24 h. Troponin I is a marker of cardiac muscle injury independent of volume status. Soldin et al. (1999) reported higher levels of troponin in children under-1 year [33]. The levels of troponin were normal and comparable in both groups. Whilst there was a rise in troponin following fluid administration at 8 h, 24 h and day 7, values were still within normal ranges and did not indicate cardiac injury.

The strength of our trial is that it includes an unselected population (except the exclusion of children with severe malnutrition, who are managed under a separate guideline [6, 34]), good adherence to protocol, and completeness of follow up. These factors plus the close clinical monitoring of children may have resulted in the low overall mortality in contrast to previous reports [11]. The low mortality (3.3%) together with the lack of other volume-specific complications indicates that slower rehydration is at least as safe as current Plan C.

The limitations include our inability to show surrogates of rehydration (time to pass urine in many of the children as routine catheterization was impractical and not acceptable to parents) and sodium endpoint (dysnatraemia at 8 h) was compromised by the high number of children with high sodium levels on admission, which we were not able to repeat as these were not done real-time but on batch processing of stored plasma at the end of the trial. Further observational studies, in Uganda in particular, are required to verify, using fresh sample analysis, whether children admitted with severe gastroenteritis frequently have hypernatraemic dehydration and whether these children had a history of taking sugary drinks. If true, then there would be a public health imperative to ensure populations are aware of and understand the risks of such practices.

## Conclusions

In this phase II trial, we have shown that slow rehydration over 8 h is as safe as WHO Plan C resulting in similar surrogate markers of patient outcome (resolution of dehydration signs, time to tolerate oral fluids and time to discharge) and no evidence of increase of adverse events (including mortality and fluid-volume related event). Training throughout the trial was required to reinforce adherence to the more complicated 2-step WHO plan C protocol. We suggest further large multicentre trials should test slow rehydration, as per GASTRO protocol, against Plan C with mortality as an endpoint.

## Data Availability

The datasets generate and analysed during the current study are available from the corresponding author on reasonable request.

## Abbreviations

AGE: Acute gastroenteritis
AVPU: Alert, voice, pain, unresponsive: system of recording patient’s level of consciousness
BIA: Bio-impedance analysis
CRF: Case report form
ETAT: Emergency triage assessment and treatment
FEAST: Fluid Expansion As a Supportive Therapy
GEMS: Global Enteric Multicentre study
GI: Gastrointestinal
ICREC: Imperial college research ethics committee
IPR: Industrial Property Act
KWTRP: KEMRI Wellcome Trust Research Programme
MRC: Medical Research Council
MUAC: Mid-upper arm circumference
NGT: Nasogastric tube
ORS: Oral rehydration solution
SERU: Scientific ethics review unit
US: Ultrasound
WHO: World Health Organization
